# Communicating cancer treatment information using the Web: utilizing the patient’s perspective in website development

**DOI:** 10.1186/s12911-014-0116-4

**Published:** 2014-12-07

**Authors:** Wendy Hopmans, Olga C Damman, Danielle RM Timmermans, Cornelis JA Haasbeek, Ben J Slotman, Suresh Senan

**Affiliations:** Department of Public and Occupational Health, EMGO+ Institute for Health and care research, VU University Medical Center, Van der Boechorststraat 7, 1081 BT Amsterdam, The Netherlands; Department of Radiation Oncology, VU University Medical Center, De Boelelaan 1117, 1007MB Amsterdam, The Netherlands

**Keywords:** Lung cancer, Patient information, Internet, Treatment decisions

## Abstract

**Background:**

Online cancer information can support patients in making treatment decisions. However, such information may not be adequately tailored to the patient’s perspective, particularly if healthcare professionals do not sufficiently engage patient groups when developing online information. We applied qualitative user testing during the development of a patient information website on stereotactic ablative radiotherapy (SABR), a new guideline-recommended curative treatment for early-stage lung cancer.

**Methods:**

We recruited 27 participants who included patients referred for SABR and their relatives. A qualitative user test of the website was performed in 18 subjects, followed by an additional evaluation by users after website redesign (N = 9). We primarily used the ‘thinking aloud’ approach and semi-structured interviewing. Qualitative data analysis was performed to assess the main findings reported by the participants.

**Results:**

Study participants preferred receiving different information that had been provided initially. Problems identified with the online information related to comprehending medical terminology, understanding the scientific evidence regarding SABR, and appreciating the side-effects associated with SABR. Following redesign of the website, participants reported fewer problems with understanding content, and some additional recommendations for better online information were identified.

**Conclusions:**

Our findings indicate that input from patients and their relatives allows for a more comprehensive and usable website for providing treatment information. Such a website can facilitate improved patient participation in treatment decision-making for cancer.

## Background

Patients increasingly wish to participate in health care decision-making [1-3]. For shared decision-making (SDM), a process in which patients are expected to use information in making well-informed decisions, their access to relevant information is crucial. Clinicians are now encouraged to integrate SDM into their routine clinical practice to support patients with cancer, and one study revealed that clinicians were generally positive with SDM [4]. Patients with cancer frequently access the Internet as a source of information [5-7], both in order to prepare for their first consultation with a specialist, and subsequently for questions arising following the consultation [8,9]. All these factors support the provision of comprehensive web-based information for patients.

However, online information may not always be adequately tailored to a patient’s perspective, particularly if professionals did not engage patient groups before developing information. Consequently, the information provided may fail to fulfil the specific information needs of patients, who may vary in health literacy. Health literacy has been defined as the degree to which individuals obtain, process, understand, and communicate about health-related information, in order to make informed health decisions [10]. Patients often have difficulties in comprehending the information provided, especially because health literacy levels are inadequate in a large part of the population [10,11].

Qualitative user testing can be used to develop information that is tailored to a patient’s perspective, using approaches that are well established in disciplines such as social sciences and marketing research [12]. Such user testing can consist of different approaches such as prompting patients to ‘think aloud’ while using online information, asking probing questions about specific areas of information, and/or asking semi-structured questions about information needs and satisfaction [13-18]. These methods allow patients to verbalize their thoughts about the information while evaluating it, thereby enabling problems such as understanding or finding information, to be identified and improved, accordingly.

The aim of the present study was to apply qualitative user testing in order to develop and evaluate online patient information for a new website on stereotactic ablative radiotherapy (SABR). SABR is a relatively recent, guideline-specified non-invasive radiotherapy treatment for patients with a stage I non-small cell lung cancer (NSCLC) [19,20]. Patients are increasingly provided with the option to undergo outpatient SABR, and should ideally also be provided with information on conventional radiotherapy and surgery. The aim of the website was to provide information about this treatment, in order to prepare patients to discuss treatment options with their physician, and ultimately to make a treatment decision. We considered this topic to provide a good opportunity for developing tailored information to patients with different levels of health literacy and educational levels.

This study focused on the following areas: (i) document the information search behaviour of patients with an early stage lung cancer prior to attending the first consultation with a radiation oncologist, (ii) determine the comprehensibility and usability of the information provided on the new website, and (iii) study the patients’ evaluation of the website. Our ultimate goal was to develop a patient-centred website that is ready to be used by patients who are eligible to undergo SABR.

## Methods

### Participants

Participants were recruited from new referrals for SABR to the Radiation Oncology department of a large university medical center, as well as their accompanying relatives. The study protocol was approved by the institutional Medical Ethic Committee (NTR 3243). Eligible patients were provided with written information about the study approximately one week prior to their first consultation, and were approached at the outpatient radiation oncology clinic by the treating physician and researcher (WH). Eligible participants had to meet the following criteria: (a) have a diagnosis of a stage I NSCLC, or be either a relative or friend accompanying the patient, (b) be scheduled to undergo SABR, (c) be a Dutch-speaking adult, (d) being able to communicate verbally, and (e) be willing to sign an informed consent form. The study was conducted between March 2012 and September 2014.

### Study design

This study consisted of three phases, namely (1) the website development, (2) a qualitative user test of the prototype website and (3) an additional user test after a redesign of the website. We included 18 participants in phase 2, and nine participants in phase 3. Previous research suggested that around 95% of usability problems could be detected by including 15-20 participants [21]. However, as phase 3 focused on partially redesigned or added sections of the website, and not on the complete website, the chosen number of 9 participants was considered appropriate [22].

### Phase 1: Website development

**Figure 1:**
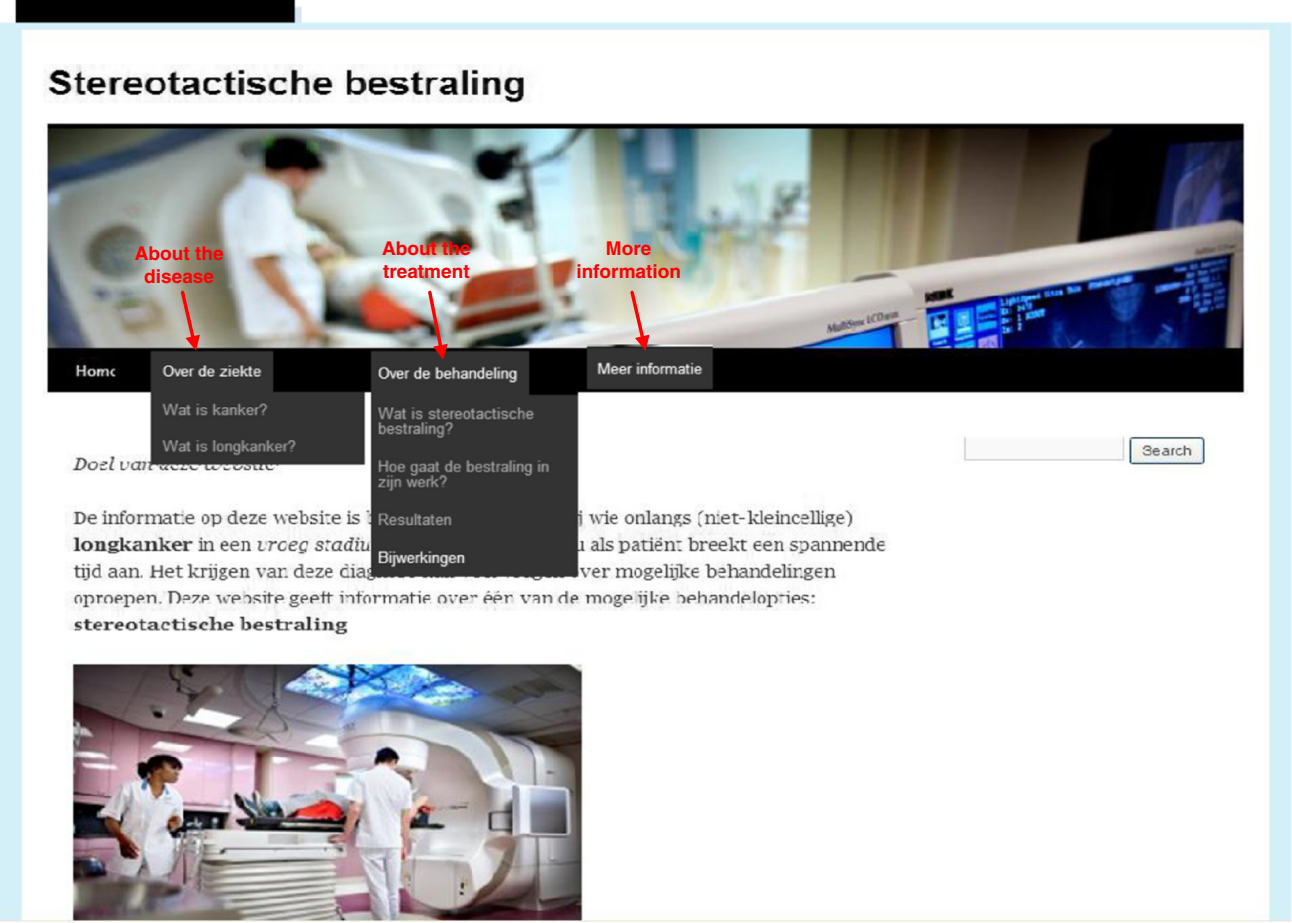
**Overview homepage with subsections.**

The prototype website was developed by a multidisciplinary research team, with assistance from a hospital information technology specialist. The research team was composed of one health scientist (WH), two psychologists (OD, DT) and two radiation oncologists (SS, CH) who held several consensus meetings about the content and design of the website. We included information about: (a) cancer in general and lung cancer, (b) details of the SABR procedure, outcomes and side-effects, and (c) references and links to related websites on cancer and its treatment. The website development was guided by patient information that had been approved by a hospital Ethics committee for a phase III trial of Either Surgery or Stereotactic Radiotherapy for Early Stage Lung Cancer (ROSEL study, ClinicalTrials.gov Identifier: NCT00687986). It was also guided by the usual information provided to all SABR patients during their first consultation at our institution with the radiation oncologist. Text, photographs and videos were incorporated in order to account for the fact that patients differ in their ability to process information in different ways [23-25]. Furthermore, textual cancer-related information with audiovisual information has been shown to improve both website satisfaction and recall of online cancer-related information in older lung cancer patients [26]. We assessed the readability of the information provided using the Flesch Reading Ease score, which scores the level of difficulty from 0 to 100 by using the length of sentences and the number of polysyllabic words [27]. We used modified formulas developed for the Dutch language by Brouwer et al. [28]. The prototype website had a Flesh Reading Ease score of 92 points, indicating that the text on the website was easily understandable by an average fifth grader. Figure 1 shows an overview of the homepage with subsections.

### Phase 2: Qualitative user test prototype website

The qualitative user test consisted of several phases. Participants provided written informed consent at the start of the interview, which was audio-taped with their permission. First, several semi-structured questions were asked to establish the participants general search behaviour on the Internet, prior to being exposed to the new website. Next, we assessed comprehensibility and usability of the website using the ‘think aloud’ approach. The participants viewed the website on a PC desktop computer in a private room in the outpatients’ clinic, and were prompted to ‘think aloud’ as they read and navigated through the website. The instruction they received was as follows: *“This is the website*http://www.stereotactische-bestraling.nl*. You may navigate through the website, just like you would do so at home. It is important that you think aloud. That means that you will try to tell me what you are thinking and doing and why you are thinking and doing that. For example, what you are clicking on, which choices you make or what stands out for you and why”*. During this phase, the researcher intervened minimally, except to say “tell me what you are thinking” when the participants were quiet. Observations about website use were recorded by scoring patients’ clicks on the website and by drawing their search track. After participants completed the website exploration, several probing questions were posed in order to determine if participants understood the main topics of the website (e.g. ‘*Can you tell me in your own words what is meant by stereotactic radiotherapy?’; “Can you tell me in your own words what these different side effects mean?”*). Finally, questions were asked in order to assess participants’ own evaluations of the website (e.g. *“What do you think of the amount of information on the website?”; “Did you miss a topic on the website?”; “What information presented on the website did you find most useful?”; ‘Would you recommend the website to others and if yes, to whom?”*). In order to be able to characterize the study population, we collected demographic details (age, sex, educational level and marital status) and assessed participants’ subjective health literacy by asking three screening questions: “How often do you have someone help you read hospital materials?”; “How confident are you filling out medical forms by yourself?” and “How often do you have problems learning about your medical condition because of difficulty understanding written information?” [29,30] In addition, we collected questions on Internet use.

### Phase 3: Redesigning the website for additional user testing

Following an analysis of participants’ verbalizations and answers to our semi-structured questions, the website content was revised with input from members of the multidisciplinary research team, all of whom subsequently reviewed and modified drafts of the new version. We then invited three patients who had previously commented on the first version of the website, together with six new participants, to ‘think aloud’ while using the redesigned website. This test phase used a similar approach as the first user test, with specific attention paid toward participants’ reactions to the redesigned and newly added sections. After the ‘thinking aloud’ part, where the participants themselves navigated through the website, we posed specific questions to participants regarding the redesigned sections (e.g. *“What do you expect to read within the subject of more information?”; “What do you expect to read within the subject of information for relatives?”; “You read about small cell lung cancer and non-small cell lung cancer, can you tell me what this means to you?”; “You read about normal radiation and stereotactic ablative radiotherapy, can you tell me what this means to you?”).* In addition, in order to be able to characterize the study population, we collected the same demographic details as in phase 2.

### Data analysis

The original audio-tapes were transcribed verbatim by the first author. Analysis consisted of various steps in analysing qualitative data [31,32]. The first author read, and re-read, all transcriptions and observation notes from phase 2, to have an initial view about how patients’ used the information on the new website. Next, two researchers (WH and OD) both independently analyzed three transcriptions. Analysis consisted of coding transcriptions into particular issues or ‘themes’ related to the 3 areas of patients’ information search prior to attending the website, the problems in comprehensibility and usability of the information, and how patients evaluated the new website. The researchers next held two consensus meetings to compare issues identified in the three transcriptions of phase 2, and to further interpret these issues. The first author analyzed the remaining transcriptions, the final results of which were discussed with the second author, before the recommendations were formulated. Finally, the first author held meetings with all members of the multidisciplinary team to discuss the findings and the recommendations to redesign the website. In addition, data derived from phase 3 were analysed by the first author and discussed with the second author to see if there were still problems in usability and comprehensibility. Next, these issues were discussed with members of the multidisciplinary team. To illustrate the quantity of how often the different issues were mentioned by our participants, we used the guidelines of Sandelowski [33]. This means that we used *most, many, often, frequently* and *generally* when a theme was mentioned by more than 75% of participants, *common* and *several* when a theme was brought up by 25% to 50% of participants, and *few* and *some* were used when a theme was stated by less than 25% of participants.

## Results

### Participants characteristics

#### Phase 2

The mean age of the 18 participants was 64.4 years (SD = 6.2), with 6 participants being relatives of patients. Six patients had low health literacy levels, and 4 had low educational levels. Nine participants accessed the Internet daily, of whom 3 indicated that they searched the Internet daily on health-related information.

#### Phase 3

The mean age of the nine participants was 66.3 years (SD = 12.1). Three participants were female, two had low health literacy levels and two low educational levels. Of these participants, seven stated that they accessed the Internet daily, and one indicated a pattern of searching daily for online health-related information (Table 1).

**Table 1:** **Sociodemograhic participant characteristics**

***Participant characteristics***		***Number of participants phase 2 (N = 18)***		***Number of “old” participants phase 3 (N = 3)***		***Number of “new” participants phase 3 (N = 6)***	
		**n**	**%**	**n**	**%**	**n**	**%**
Age	< 50	1	5.6	0	0	1	16.7
	50-64	6	33.3	0	0	1	16.7
	65-80	11	61.1	3	100.0	4	66.7
Sex	Male	10	55.6	2	66.7	4	66.7
	Female	8	44.4	1	33.3	2	33.3
Education^1^	Low	4	22.2	0	0	2	33.3
	Medium	8	44.4	1	50.0	3	50.0
	High	6	33.3	1	50.0	1	16.7
Marital status	Married	14	77.8	3	100.0	4	66.7
	Living together	2	11.1	0	0	2	33.3
	Widow	2	11.1	0	0	0	0
Health literacy^2^	Low	6	33.3	3	100.0	4	66.7
	High	12	66.7	0	0	2	33.3
Internet use	(Almost) every day	9	50.0	1	33.3	6	100.0
	Once a week	6	33.3	1	33.3	0	0
	Once a month	2	11.1	1	33.3	0	0
	Never	1	5.6	0	0	0	0
Searching for health information on the Internet	(Almost) every day	3	16.7	0	0	1	16.7
	Once a week	2	11.1	0	0	2	33.3
	Once a month	5	27.8	2	66.7	1	16.7
	Never	8	44.4	1	33.3	2	33.3

^1^Low: primary school, lower level of secondary school or lower vocational training. Medium: higher level of secondary school, or intermediate vocational training. High: higher vocational training or university.

^2^Question “Confident with forms” [29,30]: Low health literacy: patients answered: some of the time, a little of the time or none of the time. High health literacy: patients answered: all of the time, most of the time.

### General information search before seeing the website (phase 2)

Most participants indicated that they had accessed the Internet for additional information on SABR, mainly following their visit with their lung physician when the diagnosis of early stage lung cancer had been communicated. These persons mentioned using search terms in Google, such as stereotactic radiotherapy, stereotactic radiation, radiation, stereotactic, lung radiation and 4D radiation. Participants wanted to know the details of the SABR treatment procedure, possible side-effects and differences between SABR and conventional radiotherapy. Similar information search behaviour was observed for relatives. We noticed that this search for additional information was particularly apparent in those with lower educational levels and low health literacy. These participants also explicitly mentioned that physicians did provide them with information about the treatment procedure and advantages, but that this information was relatively limited and difficult to recollect during a stressful period. As one participant stated:

*“A website adds value. During the consultation, the physician tells you something and then you feel down, you have cancer. What do you remember afterwards? At home, you can take a look again at the information” (male, low health literacy, low education)*

In addition, some participants stated that they surveyed the Internet to explore whether the choice for SABR was a good decision, in case a choice had been offered between surgery and SABR.

*“I wanted to read some independent information. I did hear the whole story from my lung physician, but I wanted to know whether I made a good decision” (male, high health literacy, high education)*

### Qualitative user test prototype website (phase 2)

#### Treatment procedure

Participants appeared most interested in the webpages ‘which treatment options are available’ and ‘what is stereotactic radiotherapy’ , and indicated a preference for more detailed information on SABR as they did not completely understand the procedure. The latter was also illustrated by several questions posed about the SABR procedure during the interview:

*“All those X-rays, isn’t that bad for your body?” (female, low health literacy, medium education)*

*“So when I undergo the treatment, I won’t feel pain?” (male, low health literacy, low education)*

Both lower- and higher educated and literate participants found the information regarding the choice of number of fractions needed for SABR delivery confusing. For example, with respect to explaining the number of fractions, some participants stated that the information on the website did not match with the information from their clinician:

*“My oncologist mentioned two to three times. On the website, I read three to eight times” (male, high health literacy, high education)*

*“Usually, the treatment will take 3 to 8 times. My husband is scheduled for 12 times” (female, relative of patient, high health literacy, medium education)*

In addition, several participants appeared to be unaware of differences between conventional radiotherapy and SABR treatment.

*“Here I read that normally, radiation takes 3 to 8 times. So normally, that is the normal radiation, so how many times is stereotactic radiation then?”(male, high health literacy, high education)*

#### Participant’s understanding of epidemiological evidence

All participants had difficulty understanding concepts relating to the scientific evidence and clinical trials mentioned on the website. In this context, participants did not know what “good results” meant, and many expected to read more detailed and more exact information.

*“This section doesn’t provide much information. Only that the treatment works within patients who are fit to undergo surgery. Furthermore, not much information and the information provided isn’t very clear to me” (female, low health literacy, medium education)*

*“Can’t they just say how many patients were treated and what the results were?” (female, relative of patient, high health literacy, high education)*

#### Side-effect information

Some lower educated and less literate patients expressed an interest in having more detailed and more concrete side-effect information, e.g. how tired they would be after treatment, and what was exactly meant by radiation pneumonitis. For example:

*“Perhaps you can add an extra link or something, so you can click on it and read some extra information about all these side-effects and what to expect” (female, high health literacy, medium education)*

*“I would like to read more information about all these side-effects. They have to provide an answer, not only the names of the side-effects. How tired can you be, and what about coughing? Everyone coughs sometimes” (male, high health literacy, low education)*

#### Risk concepts

On the prototype website, we described the risk of side-effects after SABR treatment as follows: “Less than 5% of all radiation patients will have side-effects”. Not all participants clearly understood this explanation. Several patients thought that the 5% risk concerned the inconvenience of getting *one* of these side-effects, e.g. radiation pneumonia.

“*There is a 5% chance to have a radiation pneumonia” (female, relative of patient, high health literacy, medium education)*

*“What the risks are in undergoing SABR treatment are not very clear to me” (male, high health literacy, high education)*

#### Terminology

Some participants with lower educational level and low health literacy struggled with the definitions of small cell lung cancer and non-small cell lung cancer.

*“There a two different types of lung cancer. Small cell lung cancer and non-small cell lung cancer. So, that is non-small cell lung cancer, so big and small? That is not very clear to me” (male, low health literacy, low education)*

*“So, what is small cell? What is smaller than small cell”? (male, high health literacy, low education)*

The use of the device names for radiation treatment, such as “Truebeam”, “Novalis” and “Cyberknife” were often incorrectly verbalised and participants mentioned that these names “do not tell them much” and “should not be mentioned on the website”. Furthermore, a few patients also had difficulty in understanding technical terminology of treatment preparation such as a 4 Dimensional computed tomographic (4D-CT) scan, and also the fact that lung tumors move.

*“4D, that doesn’t say much to me. I can imagine that you’ll be radiated from 4 different sites” (male, high health literacy, high education)*

*“4D, so there will be 4 radiation beams on the tumor” (male, high health literacy, high education)*

*“Tumor movement? I didn’t expect that the tumor moved. I thought that the tumor was stuck on something and that it can’t move” (female, low health literacy, medium education)*

Most participants also had difficulty in understanding what was meant by “a good condition” in the context of being fit to undergoing treatment and wanted to have more concrete information.

*“That condition. I suspect that my condition needs to be checked first. What is meant by a good condition? Does that mean that I can walk regularly and feel good”? (male, high health literacy, high education)*

Despite these problems encountered, participants frequently described the definition of SABR correctly. Participants brought up terms like: very precise, directly, saving surrounding tissues, localized, high intension and involving the respiration, as illustrated by these quotes:

*“Stereotactic radiation is a very precise radiation and focuses on a small tumor. The dose of the radiation is also higher” (male, low health literacy, high education)*

*“It is a very specific radiation. The radiation focuses on the tumor without damaging the healthy tissues (male, relative of patient, high health literacy, high education)*

*“A highly intensive radiation, taking into account your breath and limit the damage to the surrounding tissues” (male, high health literacy, high education)*

*“That they only strongly radiate on the tumor itself and less on the surrounding”(female, low health literacy, low education)*

### Overall prototype website evaluation (phase 2)

When analysing the questions that were asked in order to assess participants’ own evaluations of the website (e.g., *“What do you think of the amount of information on the website?”; “Did you miss a topic on the website?”; “What information presented on the website did you find most useful?”; ‘Would you recommend the website to others and if yes, to whom?”*), the prototype website was evaluated positively by participants. Participants found the amount of information to be adequate and that the information on the treatment itself and information on side-effects were most useful:

*“The information is clear and concise” (male, low health literacy, medium education)*

*“How the treatment works. Before, I didn’t have a clue, but now I do. Especially with these pictures and videos, and how the treatment procedure is described” (male, high health literacy, high education)*

In addition, participants mentioned that they would view the website again at home and that they would also recommend it to others. Most participants felt that the website adequately informed them about SABR, and some indicated that they would have preferred to access the website prior to their first consultation with the radiation oncologist or lung physician, enabling them to ask fewer questions and to save time.

*“We did ask questions about the radiotherapy itself. How does it work, what is the procedure etc. If we would have first looked at this website, we would have already been informed. Maybe the physician would then have asked, ‘do you have any questions’? Then I would have answered: ‘we already visited the website, it is all clear’” (male, high health literacy, medium education)*

Some participants mentioned that reading information about the treatment itself, side-effects and results reduced their anxiety.

*“The information about the tumor is very reassuring and interesting. When I think of a tumor, I immediately think of a little black devil. The information here really dismantles that” (female, relative of patient, high health literacy, medium education)*

The online information was seen as complementary to the information provided by the radiation oncologist, and led to the website information being trusted by participants. Furthermore, some patients had a preference for additional information on their role as patients, such as on how to obtain a second opinion, on contacting patient organizations, and on where to go in case of questions. A final topic raised by some participants was that they would prefer a website providing additional information about surgery.

*“I would prefer more information about the other treatment option on the website as well. Then you would have it all in one” (male, low health literacy, high education)*

*“I would prefer a sort of checklist, with both information about surgery and radiation. Then the choice might be easier to make” (male, high health literacy, high education)*

### Redesigning the website for additional user testing (phase 3)

**Figure 2:**
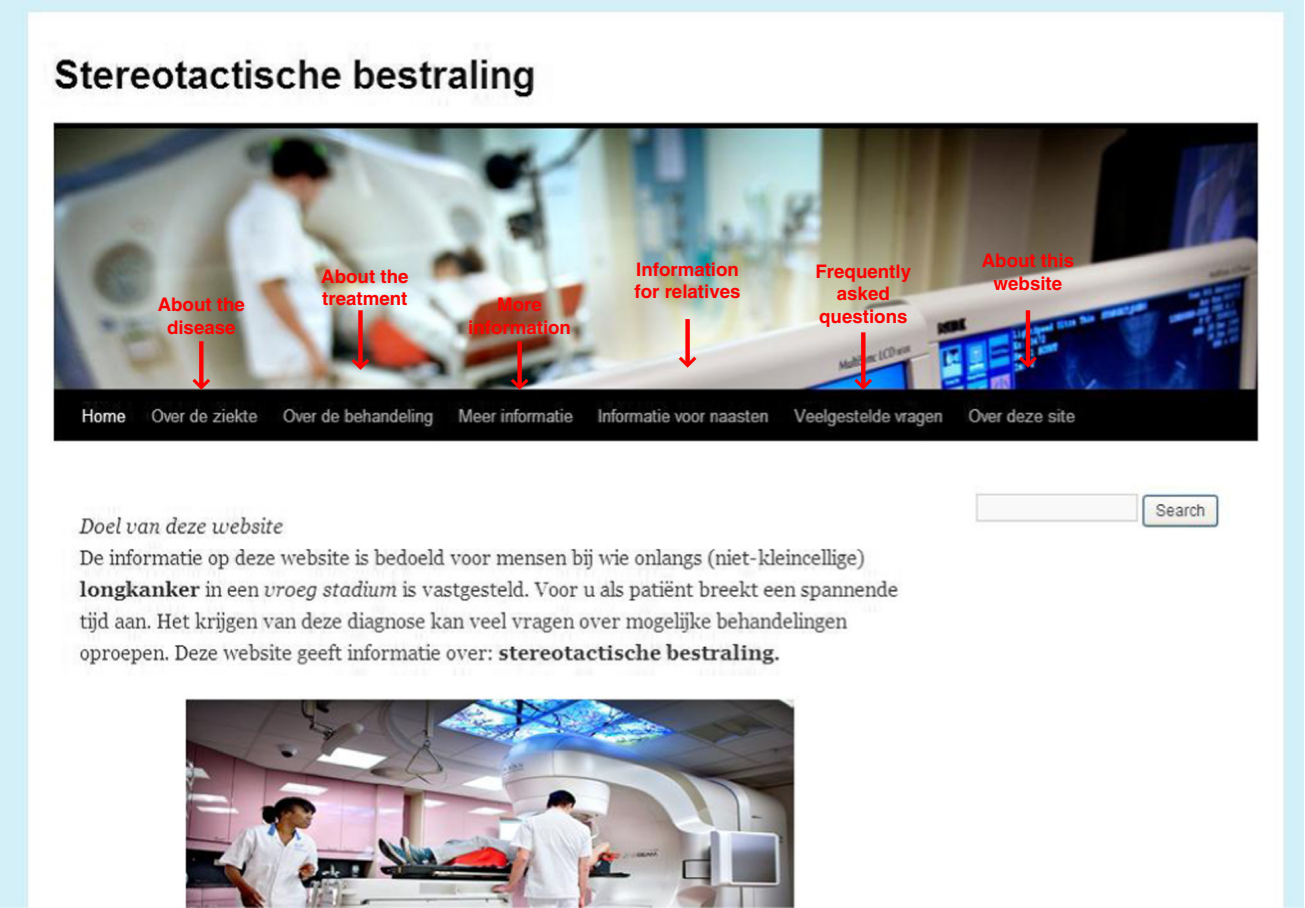
**Overview redesigned homepage with subsections.**

Table 2 summarizes several of the changes made to the website, and Figure 2 displays an overview of the redesigned homepage with sections. The revised website included more detailed information about the treatment process, results and side-effects, including additional photographs and videos. For example, we added an information video about the 4D-CT scan, and included a table comparing features on both conventional radiation and SABR. Potential side-effects of SABR were also explained more thoroughly, and the description of risk information was reworded. We changed this description from a percentage (e.g., less than 5% of all radiation patients will have side-effects) to a proportion (e.g., 1 out of 20 patients that undergo this treatment, suffers from side-effects). We also included three new sections with “information for relatives”, “frequently asked questions”, and “about this website”, in response to the information needs of patients (Table 2 and Figure 2). The redesigned website was tested again among users. The information video on the 4D-CT scan procedure, and the table comparing conventional radiation therapy and SABR, were understood well and evaluated positively.

**Table 2:** **Changes made to the website**

**Website section**	**Prototype website**	**Final website**
*About the disease*	Information about the incidence of lung cancer in the Netherlands.	We added two web sources with information for patients:
		- Cancer in the Netherlands, trend and prognoses
		- Integral Cancer Centre, The Netherlands
*What is cancer?*	Description of cell division and how lung cancer occurs.	We added an information video titled: “What is cancer”. http://www.youtube.com/watch?feature=player_embedded&v=L2uF0qk1jck
*What is lung cancer?*	Non-small cell lung cancer and small-cell lung cancer are mentioned. Furthermore, the different stages are explained.	We added information about the two types of lung cancer and explained the differences more thoroughly:
		“Small cell lung cancer is a fast growing type of lung cancer. It spreads more quickly to the lymph nodes, and is therefore almost always treated with chemotherapy or a combination of chemotherapy and radiotherapy. Patients with small cell lung cancer are not candidates for stereotactic ablative radiotherapy”.
		“Non-small cell lung cancer is the most common sub-type of lung cancer. It usually grows and spreads more slowly than small-cell lung cancer”.
*Which treatment options do I have?*	Description of the different treatment options for an early stage lung cancer (surgery and stereotactic ablative radiotherapy) and information about the importance of a good condition.	- We added information about the importance of being in a good condition. - We mentioned that the condition is tested, for example by a bicycle-test, to provide some meaning to the term ‘good condition’.
*What is stereotactic ablative radiotherapy?*	Description of stereotactic ablative radiotherapy (precision radiation etc) and the treatment process.	- We added information about both stereotactic ablative radiotherapy and conventional radiotherapy. A table was added so that patients could easily see differences between these two radiation approaches.
		- We revised the text and described the treatment itself more thoroughly, with added information about the treatment team (radiotherapist etc).
		- We added an information video titled: “What is stereotactic ablative radiotherapy”. http://www.youtube.com/watch?feature=player_embedded&v=kdrJsyl5EPg
*What happens during treatment?*	Description of the radiation preparation, including a 4D-CT scan, drawing marks on the body, x-rays being made etc.	- We added about a 4D-CT scans, including an information video titled: “4D-CT scan and radiation”.
		- We added information about the radiation preparation and added two new photographs of marks/lines drawn on the body surface to assist in positioning.
		-Also added was a section with information about radiation delivery, and described the whole process from arriving at the hospital until going home after treatment.
*Results*	Description of the results of SABR treatment	- We revised this information for easier understanding by patients: “Until now, this way of radiotherapy is particularly done within patients who were not a candidate to undergo surgery. This could be because they were not fit enough. In such patients, good results have been achieved, and the likelihood of being cured is the same as when a patient undergoes an operation. As a result of these good results, stereotactic radiotherapy is increasingly used in patients who are a candidate to undergo surgery, but who declined to undergo surgery. Reasons for the latter included an aversion to possible side-effects of surgery, or because of a patients preference for stereotactic radiotherapy”.
		- We added references to different studies which had been performed using stereotactic ablative radiotherapy.
*Side-effects*	The different side-effects are mentioned as follows: Some patients experience:	- We extended information about the potential side-effects. For example:
		*Some discomfort lying in the machine:* The couch of the machine has a hard surface, which can make it uncomfortable for patients.
	• Some discomfort lying in the machine	*Fatigue:* Many patients experience tiredness, which may partly be due to travelling to and from the hospital. To minimize this, patients should plan for a less hectic schedule during treatment.
	• Fatigue	
	• Painful ribs after treatment	
	Etc.	
	Risk information was provided as follows: “During the treatment itself hardly any side effects occur. Less than 5% of all patients experience these side effects”	The information on risks was presented differently: “At the very most, 1 out of the 20 patients who undergo this treatment, will have side-effects”.
*More information*	This section provides information when patients still have some questions about stereotactic radiotherapy after reading the information on the website. Patients are advised to contact their physician.	- We added information about obtaining a second opinion, and included a link to a website with information about these second opinions.
		-We added an information video, titled: “Conversation with your doctor”. http://www.youtube.com/watch?feature=player_embedded&v=6Tlb36jni64
*Information for relatives*	-	-A new section was added to cater to the information needs of relatives.
		-We added an information video titled: “Relatives of cancer patients”. http://www.youtube.com/watch?feature=player_embedded&v=75btAnXrq6A
*Frequently asked questions*	-	We added a new section with frequently asked questions during interviews, e.g.:
		“Could the additional x-rays performed be bad for one’s health?”
		“Does the treatment hurt?”
		“Do I remain radioactive following the treatment?”
*About this website*	-	- A new section was added with information about the aims of the website.
		- We added website links and references.

*(Reaction to 4D-CT scan video): “Now you can imagine how you have to lay down and you can see the radiation going through your body, right on the tumor cell” (male, high health literacy, medium education)*

*(Reaction to comparing table): “Stereotactic radiation is very precise, on the tumor cell itself, and you only have to be here for a few times. This is not the case with conventional radiation, which takes much longer and causes more damage” (female, high health literacy, medium education)*

However, the extra focus on conventional radiotherapy led to some participants questioning why this form of radiotherapy was not considered a treatment option for them. Analysis of participants’ reactions to the reformulated explanations of small cell lung cancer and non-small cell lung cancer, as well as additional information on side-effects, suggested that the explanations provided on the website were comprehensible.

*“Small cell lung cancer is the bad one, that goes very fast. Then the cancer has spread through your whole body. I have non-small cell lung cancer, that is still good, it is still small, and it is treatable” (female, high health literacy, high education)*

*“Radiation pneumonitis. Yes, that is because of the radiation. It can damage your lung and then your lung gets infected” (female, high health literacy, medium education)*

However, despite rewording of the text on risks (e.g., that 1 out of 20 patients that undergo this treatment, suffers from side-effects, instead of less than 5% of all radiation patients will have side-effects), this aspect was not correctly understood by all participants:

*“Well, that’s 20%. Personally, I would recommend to describe 20%, but not everyone is good at mental arithmetic” (male, high health literacy, low education)*

*“1 out of 20 patients? That is quite a lot. That is 20%” (male, high health literacy, high education)*

When asked about their expectations of the nature of “more information” (e.g., this section provides information when patients still have some questions about SABR after reading the information on the website, patients are advised to contact their physician), participants did not always know what to expect:

*“I’ve got no idea. Maybe more information about the hospital itself?” (female, high health literacy, medium education)*

*“I’ve got no clue, perhaps information about after care?” (male, low health literacy, low education)*

This suggests that this section might better be reworded on the website to provide it some meaning, for example ‘conversation with your doctor’.

The revised and expanded sections appeared clearer for patients, and were considered useful as indicated by the comments, for example:

(Reaction to the section “information for relatives”): *“That I can imagine what my partner goes through. I’m the one with the cancer, but he is also. He sympathizes, thinks along, stands by me, complements me, and often he knows more than I do” (female, high health literacy, medium education)*

Overall, the redesigned website was considered more comprehensible and usable.

*“The information is accessible, easy to understand and my relatives can also benefit from this. So when I want to explain something about the treatment, I can recommend them to access the website” (male, low health literacy, low education)*

*“I think it is a very good website, especially the combination of videos and text. Videos make it more concrete because of the fact that you now know what to expect and text is pleasant because it explains everything briefly with additional background information” (female, high health literacy, high education)*

Participants succeeded in navigating the website in a logical manner, and the majority identified no further areas for improvement. However, one participant suggsted that patient stories might prove a useful addition to the website. None of the participants reported missing information.

*“Did I miss anything? No, I don’t think so. It is clear, looks good. I’m going to view the website again at home. What was the name of the website again?” (male, high health literacy, high education)*

## Discussion

Since its introduction in the early 1990’s, the growing use of SABR for stage I NSCLC has resulted in improved population-based survival rates [34,35]. As more patients are now eligible to undergo SABR, we considered SABR an ideal indication for developing online information tailored to the patient’s perspective. Our study used qualitative user testing in order to develop online information about SABR, and a key finding was that our website created by experts was not considered ideal by patients and their relatives. Many participants wished to have different information than what was provided initially on the website, especially more detailed information about SABR and side-effects. In addition, patients did not fully understand the medical terminology used, or the information used to describe the scientific evidence regarding SABR.

The participants’ felt a need for detailed and concrete information that had not been provided on the initial website. Specifically, participants wanted a step-by-step description of details of the SABR treatment procedure itself, as well as detailed information about the side-effects of SABR. These finding are consistent with previous work which found that older cancer patients placed ‘treatment-related information’ most important [36]. Our finding suggests that routine patient information now provided by thoracic oncologists may not be optimal.

To the best of our knowledge, no other studies have been conducted on the information needs of patients facing a new treatment option for cancer. However, studies on more conventional treatment options showed that information provided by clinicians about treatment options and outcomes is often considered to be insufficient by patients [37-40]. In addition, even when clinicians do provide extensive information, patients and relatives may still want to review the information at home. The latter was explicitly stated by some of our participants with low health literacy and low educational levels. Informing patients about a relevant website, both before and after their first consultation seems essential in supporting patients in asking specific questions, preparing them for discussions with their clinicians and sharing their concerns, as well as in supporting their treatment decisions.

We identified problems in patients understanding of the medical terminology provided, such as the definition of NSCLC and terms like ‘4D-CT scan’ , a finding reported in earlier studies, for example, on the comprehension of both verbal and written patient information [41-44]. A scheme developed to classify errors in lay comprehension of medical information, identified ‘clinical concepts’ and ‘terminology’ as essential categories of lay errors in understanding medical documents [45]. As clinicians do use these terms in their consultations, it appears important to provide clear explanations of these terms consistent in online communication. These findings suggest that clinicians and health professionals often overestimate patients’ health literacy and use medical terms with the presumption that a patient understands it [46,47]. Our qualitative assessment showed that lay errors in comprehending web information can be relatively easily identified and solved, for example by providing extra explanations.

Another common problem identified concerns misunderstanding of information relating to the scientific evidence with respect to treatment options. Patients have difficulties in understanding information pertaining to clinical research, and treatment risks and benefits are particularly poorly understood [48]. Patients have particular trouble understanding risk information, which is typically complex and abstract in nature [49,50], and which impacts on patients’ comprehension, as shown in our study, as well as by others [51,52]. Difficulty in understanding risk information may persist, even when information is presented in accordance with current guidelines [49]. Consequently, use of visual aids (i.e., icon array or bar graph) may be necessary to improve understanding of risk information [53].

One limitation of our study which must be acknowledged is that our study population was already referred to a department of Radiation Oncology. As such, our conclusions may be less applicable to the larger population of patients with early stage lung cancer, for example those who may be receiving other treatment advise.

## Conclusions

By using qualitative user testing, we identified problems faced by participants in interpreting available online information about SABR, an outpatient treatment for early-stage lung cancer. Our study patients and their relatives desired information on areas that were not foreseen by professionals who developed the website. These findings indicate that a careful testing of websites among the targeted patient groups is necessary in order to develop patient-centred information that patients may use for treatment decision-making.

## Abbreviations

SABR: Stereotactic ablative radiotherapy
SDM: Shared decision making
NSCLC: Non-small cell lung cancer
